# Recognition and management of left atrial dissection during mitral repair

**DOI:** 10.1186/s13019-024-02641-x

**Published:** 2024-03-19

**Authors:** Haya Alshaabi, Jack F. Donaghue, Denise M. Franko, Jock N. McCullough

**Affiliations:** 1 Geisel School of Medicine at Dartmouth, Hanover, NH USA; 2 Dartmouth-Hitchcock Medical Center, Lebanon, NH USA

**Keywords:** Left atrial dissection (LAtD), Mitral valve surgery, Transesophageal echocardiogram (TEE)

## Abstract

**Background:**

Left atrial dissection (LAtD) is a rare but potentially life-threatening complication of mitral valve surgery. Its management is not well stablished in the literature. However, early recognition through intraoperative TEE and attention to changes in the left atrial free wall during saline leak testing can lead to avoidance of severe complications.

**Case presentation:**

We report a case of LAtD detected by intraoperative transesophageal echocardiogram (TEE) following mitral valve repair for primary mitral valve regurgitation secondary to degenerative mitral valve disease with MAZE IV procedure for atrial fibrillation. LAtD was noted on TEE as an expanding double density along the wall of the left atrium with a jet originating at the posterior annulus flowing into the LAtD which was repaired. Separation from bypass following LAtD repair was complicated by severe biventricular dysfunction requiring significant inotropic support and placement of an intra-aortic balloon pump (IABP). Patient’s post-operative course was further complicated by right sided heart failure requiring placement of a right sided impella which was subsequently removed on POD 4. Patient was discharged home on POD 17. Transthoracic echo at 1 month, 3 months demonstrated resolution of the LAtD. A follow up echo at 4 years showed complete resolution of the LAtD with an intact mitral repair, trace mitral regurgitation, and a mean gradient across the repair of 3 mm Hg.

**Conclusions:**

Left atrial dissection is a rare but serious complication of mitral valve surgery. We provide a review of the current literature regarding LAtD, emphasizing the need to consider this complication early during mitral surgery to allow for uncomplicated repair.

## Background

Left atrial dissection (LAtD) is a rare but potentially devastating complication of mitral valve procedures [1]. The pathoanatomy represents an iatrogenic separation of the layers of the left atrial wall resulting in a false lumen between the endocardium and epicardium. It often originates at the posterior mitral annulus [2]. The etiology is not fully understood, but predisposing factors include severe calcification of the mitral annulus requiring extensive intraoperative debridement, excessive traction on sutures, oversizing of the implant and inadequate reversal of anticoagulation [1–3].

The diagnosis can be observed intraoperatively or in the early perioperative interval but has been reported as late as 20 years after surgery [1–3]. The diagnosis is typically confirmed by transesophageal echocardiography (TEE) [4]. Urgent surgical intervention is often necessary to repair the dissected atrial wall and prevent further complications including free rupture into the pericardiac space. There are several case reports of left atrial dissection in the literature, yet the optimal management strategy remains unclear. Here we present a case where the diagnosis was made intraoperatively. Our goal is to remind all surgeons of this very rare and potentially devastating complication as well as how to recognize it early when repair is simple.

## Case presentation

A 71-year-old male with a history of atrial fibrillation and degenerative mitral valve disease with severe mitral regurgitation (MR) presented for mitral valve repair and MAZE procedure.

His preoperative echo revealed bi-leaflet mitral valve prolapse with severe regurgitation. His left atrium was severely dilated (80 ml/m2 volume index) but his right and left ventricular function were normal. His pre-operative cath showed right coronary dominance with no evidence of pulmonary hypertension or obstructive cornornary artery disease (CAD).

A standard biatrial MAZE IV procedure was performed.

**Fig. 1:**
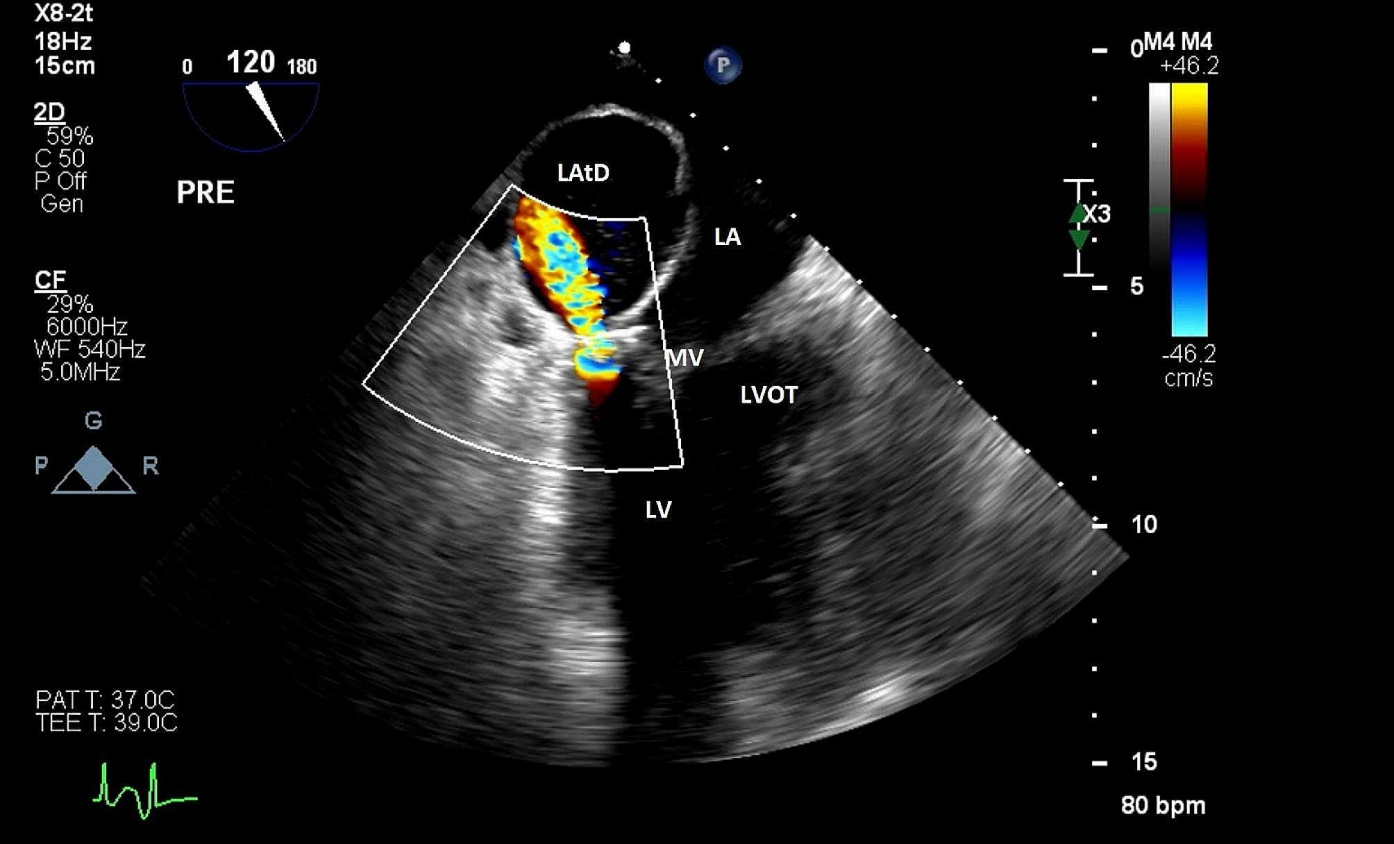
Rapidly expanding LAtD following initial wean from cardiopulmonary bypass. Mid esophageal LAX

Repair of the mitral valve was perfomed through a left atriotomy after fully developing Wattersons groove. The repair included a quadrangular resection of a flail P2 segment, sliding plasty and a 38 mm posterior annuloplasty band. After removal of the cross clamp, during partial bypass, an expanding double density along the wall of the left atrium was noted on TEE with a jet originating at the posterior annulus flowing into the LAtD. (Fig. 1). Bypass was resumed and the left atrium was re-opened. The annuloplasty band was removed and the posterior leaflet was taken off the annulus allowing the visualization of a 4 mm defect at the LV-LA annular junction below the P3 leaflet. This communication was closed with a single pledgeted mattress suture and the posterior leaflet repair was re-aproximated to the annulus. A new 36 mm annuloplasty band was placed and the left atrium was reclosed.

**Fig. 2:**
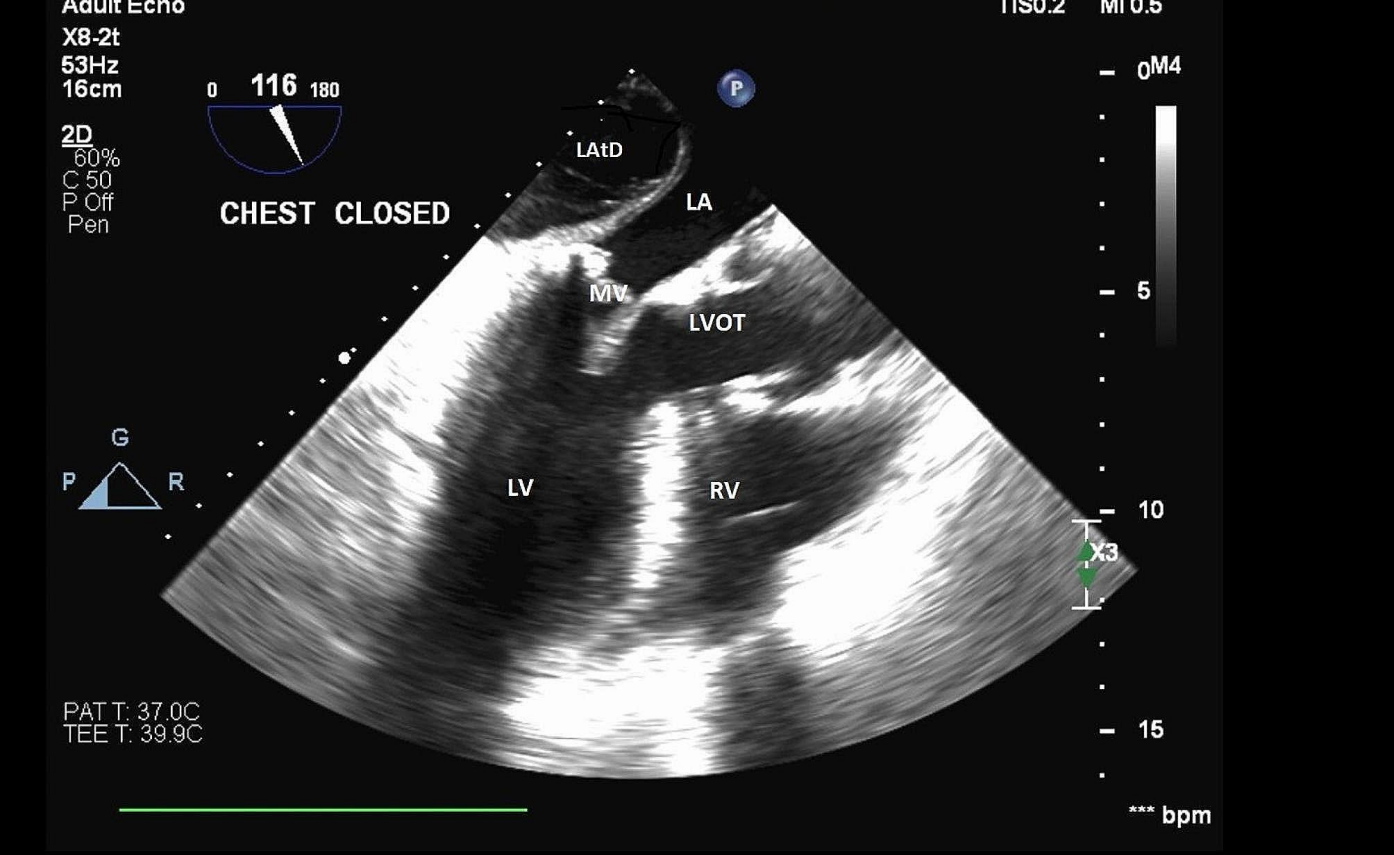
Dissection pocket of unchanged size at the end of surgery with noted hematoma formation. Mid esophageal LAX

Separation from bypass was complicated by severe biventricular dysfunction requiring significant inotropic support and placement of an intra-aortic balloon pump (IABP). TEE on chest closure (Fig. 2) was notable for a non-expanding dissection pocket with hematoma formation. There was trace residual mitral regurgitation.

On post operative day 2 (POD2), a right sided Impella was deployed for cardiogenic shock secondary to right ventricular failure. TEE demonstrated no flow into the LAtD and an intact mitral repair.

**Fig. 3:**
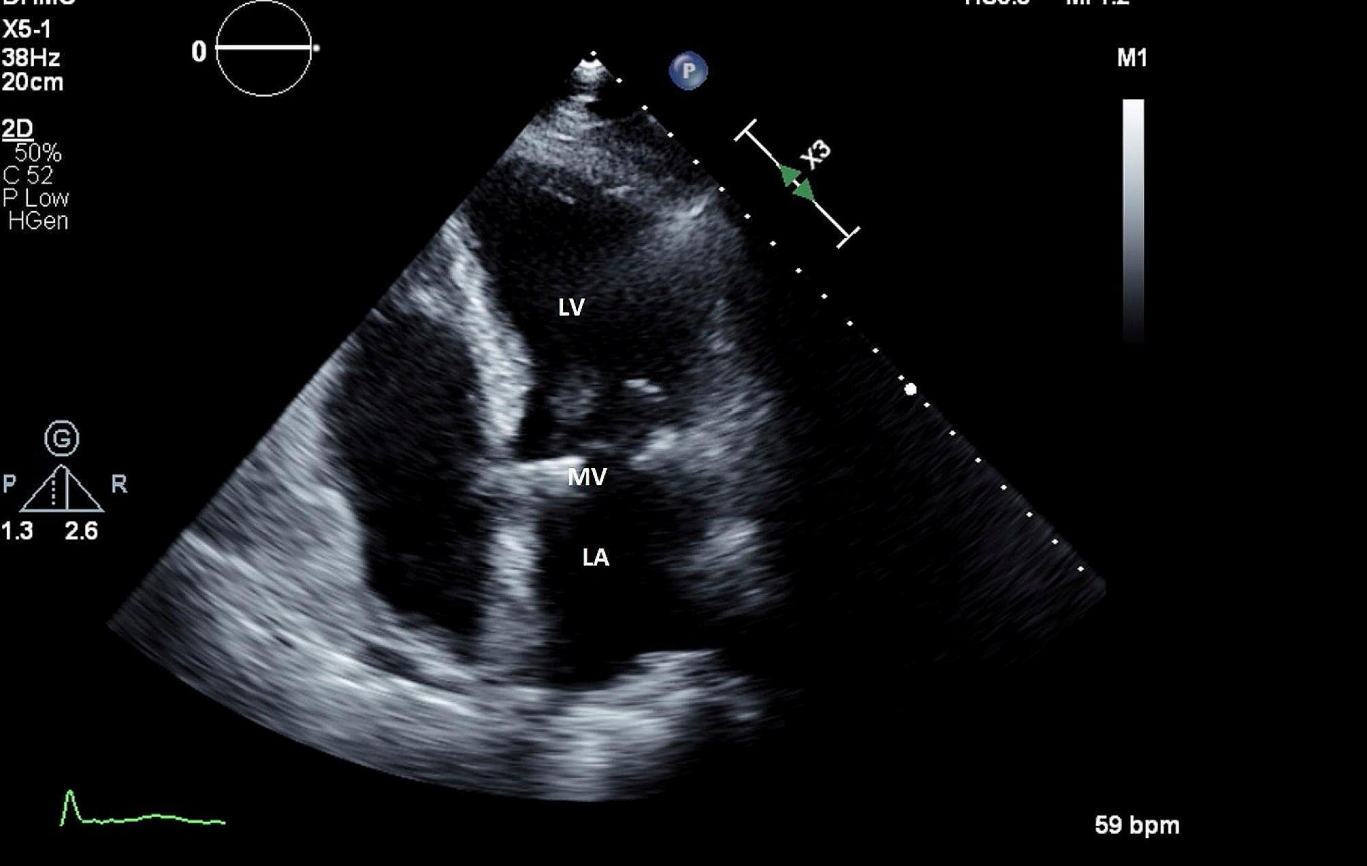
Follow-up echo at 1 month demonstrating resolution of atrial dissection

The patient’s hemodynamics improved over the following 4 days allowing removal of the Impella and IABP. He was discharged home on POD 17. Transthoracic echo at 1 month, 3 months demonstrated resolution of the LAtD (Fig. 3). A follow up echo at 4 years showed complete resolution of the LAtD with an intact mitral repair, trace mitral regurgitation, and a mean gradient across the repair of 3 mm Hg.

## Discussion and conclusions

LAtD can be noted intraoperatively or years after surgery [5–7]. The differential diagnosis of postoperative hemodynamic instability includes cardiac tamponade, valve failure, myocardial ischemia, arrhythmias, and many other complications, all significantly more common than LAtD. Early TEE can identify many of the causes of post operative hemodynamic instability. Findings consistent with left atrial dissection on TEE include duplication of the left atrial wall, significant displacement of the dissected cardiac wall and flow between the false cavity created by dissection and a cardiac chamber [4, 7, 8].

Treatment options for LAtD include close observation or surgery to evacuate the hematoma and obliterate the false cavity and entry point [3]. Selection of the most appropriate management approach depends on the patient’s hemodynamic stability. A previous review of 89 left atrial dissection cases reports no survival benefit to surgical management over conservative management in hemodynamically stable patients [1], however, only 25% of these cases were identified intraoperatively. In our case, given the visualized rapid expansion of the dissection, the patient was managed with immediate return to bypass, identification of the entry site and its closure followed by re repair of the valve.

LAtD causes hemodynamic instability by diminishing cardiac output, mainly through obstruction of mitral valve inflow or pulmonary vein orifice [5, 8]. The persistence of our patient’s hemodynamic instability and its progression to cardiogenic shock despite early recognition and repair of his LAtD was secondary to severe distributive shock, persistent vasoplegia and RV dysfunction likely the result of difficulty protecting the right ventricle during a prolonged bypass run relying on retrograde cardioplegia. In our case, post operative RV impella support was initiated with good effect allowing for diuresis and rapid weaning of vasoactive support. The Impella was successfully removed on POD4. TTE at 1 month demonstrated complete resolution of LAtD but was notable for a mildly reduced ejection fraction (49%) with mildly dilated right ventricle.

After this case, the senior author was performing a mitral valve repair which included a sliding plasty. During saline “leak testing” he noticed the beginning of an interatrial dissection manifested by discoloration and ballooning of the left atrial wall just below the mitral annulus. The repair was taken down and a communication from the left ventricle into the potential space was noted along the suture line joining the advanced P3 segment and the underlying mitral annulus. A single pledgeted mattress suture closed the defect, and the repair was reconstructed. The patient was separated from bypass without difficulty. His intraoperative TEE showed no residual mitral regurgitation and no evidence of dissection of the left atrial wall. The senior author’s experience in the case described above allowed early identification and repair of LAtD prior to the development of a significant dissection.

Furthermore, the avoidance of postoperative anticoagulants has been recommended to prevent complications post LAtD and support conservative management [9]. Our patient was placed on warfarin anticoagulation given his history of persistent atrial fibrillation. Despite the use of warfarin postoperatively he had complete and stable resolution of his dissection following closure of the entry tear.

In conclusion, left atrial dissection is a rare but serious complication of mitral valve repair. We hope that surgeons operating on the mitral valve remain aware of this complication and recognize it early when repair is straightforward.

## Data Availability

Patient’s case was reviewed using Dartmouth Hitchcock Medical Center electronic records system. No identifiable health information is included in our case report. No datasets were generated or analysed.

## Abbreviations

LAtD: Left atrial dissection
TEE: Transesophageal echocardiogram
MR: Mitral regurgitation
LV: Left ventricule
LA: Left atria
IABP: Intra-aortic balloon pump
